# Cytotoxicity assessment of Bio-C Repair Íon+: A new calcium silicate-based cement

**DOI:** 10.34172/joddd.2021.026

**Published:** 2021-08-25

**Authors:** Celso Afonso Klein-Junior, Roberto Zimmer, Tãnyre Dobler, Vanessa Oliveira, Daniel Rodrigo Marinowic, Ahmet Özkömür, Eduardo Galia Reston

**Affiliations:** ^1^Department of Operative Dentistry, School of Dentistry, Lutheran University of Brazil, Canoas, RS, Brazil; ^2^Department of Operative Dentistry, School of Dentistry, Lutheran University of Brazil, Cachoeira do Sul, RS, Brazil; ^3^Brain Institute, Neuroscience laboratory, Pontifical Catholic University of Rio Grande do Sul, Porto Alegre, RS, Brazil

**Keywords:** Calcium silicate, Cell survival, Fibroblasts, Mineral trioxide aggregate

## Abstract

**Background.** Direct pulp capping is a method designed to preserve the exposed dental pulp. Due to good biological, physical, and mechanical properties, new versions of calcium silicate-based materials have been developed as pulp capping materials. The present study aimed to evaluate the cytotoxic effects of four calcium silicate-based pulp capping materials, of which the Bio-C Repair Íon+ is still in an experimental phase.

**Methods.** Biodentine, MTA Repair HP, Bio-C Repair, and Bio-C Repair Íon+ cements were dispensed in a metallic matrix to produce 125-mm^3^ specimens, which were immersed in Dulbecco’s Modified Eagle Medium (DMEM) to obtain extracts. NIH 3T3 cells were cultured and exposed to the extracts for 24 hours and seven days. Cell viability was assessed by the *methyl tetrazolium* test (MTT). The mean values for the experimental and control groups (without treatment) were compared by analysis of variance (ANOVA) and post hoc Tukey tests, considering a significance level of 5%.

**Results.** All the tested materials demonstrated a reduction in cell viability (*P* < 0.05). According to ISO 10993-5: 2009 (E), Bio-C Repair Íon+ exhibited mild and moderate cytotoxicity in the 24- hour and 7-day analyses, respectively. Bio-C Repair and Biodentine showed mild cytotoxicity, and MTA Repair HP exhibited moderate cytotoxicity at both intervals.

**Conclusion.** The highest cell viability was demonstrated by Biodentine, MTA, and Repair HP, in descending order. Bio-C Repair and Bio-C Repair Íon+ showed moderate cytotoxicity, similar to MTA Repair HP in the 7-day analysis.

## Introduction

Advances in dentistry are associated with advances in dental materials and novel therapies that promote tissue regeneration without affecting the cell signaling processes mediated by undifferentiated mesenchymal cells.^1^ Direct pulp capping is a method designed to preserve the exposed dental pulp with a protective agent, inducing hard tissue repair.^2^ The protective materials used must have adequate biocompatibility and bioactivity to promote stem cell activity and pulp healing in permanent teeth.^3^

For many years, the material of choice for pulp exposure cases was calcium hydroxide until studies demonstrated better clinical performance of calcium silicate-based materials, such as mineral trioxide aggregate (MTA).^4,5^ Both materials have a similar mechanism of action; however, calcium silicate-based cements induce the formation of a more uniform and thick mineralized barrier, in addition to causing less inflammatory response and less pulp tissue necrosis.^6^

MTA cement has hydrophilic properties, good radiopacity, low solubility, high pH, antimicrobial activity, and the ability to expand after insertion in the place of interest.^7-9^ However, the material is difficult to handle for insertion and condensation in the cavity and has a long setting time.^10,11^ Due to good biological, physical, and mechanical properties, new versions of calcium silicate-based materials have been developed for use as capping materials.

To overcome the weaknesses of the MTA, Biodentine was marketed as a dental substitute.^12,13^ This material can be used similar to MTA, with similar indications, to induce the formation of a mineralized barrier in cases of pulp capping.^14^ In addition, it has the advantage of reducing the setting time, ease of handling, and better mechanical and biological properties.^15,16^

Following the idea of simplifying the operative technique, Bio-C Repair, a ready-to-use bioceramic repair material, was developed. This recently introduced material has shown cytocompatibility results similar to MTA, in addition to being biocompatible and inducing biomineralization.^17^ Although calcium silicate-based bioceramic compositions can release calcium ions, the presence of metal ions promotes greater bioactivity and induces complete regeneration and repair.^18^ Therefore, still in the experimental phase, Bio-C Repair Íon+ is a cement with metallic particles in its composition. So far, this material has not been studied in vitro and in vivo, comparing its cytocompatibility with MTA Repair HP and Biodentine, which are materials widely used directly in pulp tissue. Therefore, the present study aimed to evaluate the toxic effects of four pulp capping calcium silicate-based cements on NIH 3T3 cells, of which the Bio-C Repair Íon+ is still in an experimental phase.

## Methods

In the present *in vitro* study, four repair cements were used: Bio-C Repair Íon+ (Angelus, Londrina, PR, Brazil), Biodentine (Septodont, St-Maur-déss-Fosses, France), Bio-C Repair (Angelus, Londrina, PR, Brazil), and MTA Repair HP (Angelus, Londrina, PR, Brazil) (Table 1). Necessary cement quantities were dispensed inside a metallic matrix measuring 8 mm in diameter and 5 mm in thickness to produce specimens (n = 5) of 125 mm^3^.

**Table 1:** Calcium silicate-based cements used and their compositions, setting times, and manufacturers

**Cement**	**Composition**	**Setting time (min)**	**Manufacturer**
Bio-C Repair	CaSiO_3_, CaAl_2_O_4_, CaO, ZrO_2_, Fe_2_O_3_, SiO_2,_ and dispersing agent.	≤120	Angelus
Bio-C Repair Íon+	CaSiO_3,_ MgO_3_Si, CaO, ZrO_2_, SiO_2_, setting agents, and dispersing agent.	≤120	Angelus
Biodentine	Powder: 3CaO.SiO_2_, ZrO_2_, CaO, CaCO_3_, yellow pigment, red pigment, and Fe_2_O_3_ brown. Liquid: CaCl_2_.2H_2_O, hydrosoluble polymer, and purified H_2_O.	12	Septodont
MTA Repair HP	Powder: 3CaO.SiO_2_, 2CaO.SiO_2_ 3CaO.Al_2_, CaO, CaWO_4_. Liquid: H_2_O and plasticizer.	15	Angelus

Abbreviations: 3CaO.SiO_2_: tricalcium silicate; ZrO_2_: zirconium oxide; CaO: calcium oxide; CaCO_3_: calcium carbonate; Fe_2_O_3_: iron oxide; CaCl_2_.2H_2_O: calcium chloride dihydrate; H_2_O: water; 2CaO.SiO_2_: dicalcium silicate; 3CaO.Al_2_: tricalcium aluminate; CaWO_4_: calcium tungstate;_;_ CaSiO_3_: calcium silicate; CaAl_2_O_4_: calcium aluminate; MgO_3_Si: magnesium silicate; SiO_2_: silicon dioxide.

### Cell culture

The cells used in the present study were NIH/3T3 mouse fibroblasts (ATCC®-American Type Culture Collection-TCC, Old Town, Maryland, USA) cultured in Dulbecco’s Modified Eagle Medium (DMEM; Invitrogen®, Carlsbad, California, USA). This medium was supplemented with 10% fetal bovine serum, 100 U/mL of penicillin (Gibco, Grand Island, NY, USA), 100 U/mL of streptomycin (Gibco, Grand Island, NY, USA), and 100 μg/mL of gentamycin (Gibco, Grand Island, NY, USA). The cells were kept in a humidified incubator at 37ºC under 5% CO_2_.

### Cell viability

The samples were sterilized by ethylene oxide (Esteriliplus, Porto Alegre, Rio Grande do Sul, Brazil) and immersed in DMEM medium. The surface area of the specimen for the average volume ratio was 3 cm^2^/mL, according to ISO 10993-12 (2012).^19^ The area was calculated based on the total dimensions of the specimen, disregarding porosity. The extracts were tested for cell viability after 24 hours and seven days in the incubator.

Cell viability was assessed by analyzing mitochondrial activity using the *methyl tetrazolium* test (MTT) technique. The extracts were placed in contact with the cell culture, as described by Klein-Junior et al.^20^ The percentage of viable cells was calculated compared to the negative control, where the cells received no treatment, and the cytotoxicity grade was classified according to ISO 10993-5 (E) (Table 2).

**Table 2:** Grade of cytotoxicity according to with ISO 10993-5:2009 (E)

**Grade**	**Reaction**	**Culture conditions**
0	None	No cell lysis, no reduction in cell growth
1	Slight	Not more than 20% reduction in cell growth
2	Mild	Not more than 50% reduction in cell growth
3	Moderate	Not more than 70% reduction in cell growth
4	Severe	Nearly complete or complete destruction of cell layers

### Statistical analysis

The normal distribution of the variables was tested using the Shapiro-Wilk test. The mean values determined for the experimental groups for cell viability were compared using one-way analysis of variance (ANOVA), followed by post hoc Tukey tests. The analyses were carried out at a significance level of 5%.

## Results

**Figure 1:**
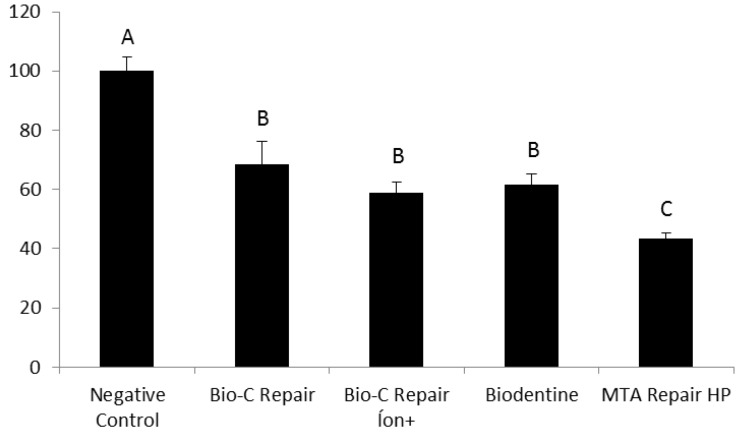
Cell viability of calcium silicate-based cements in the 24-hour analysis.

**Figure 2:**
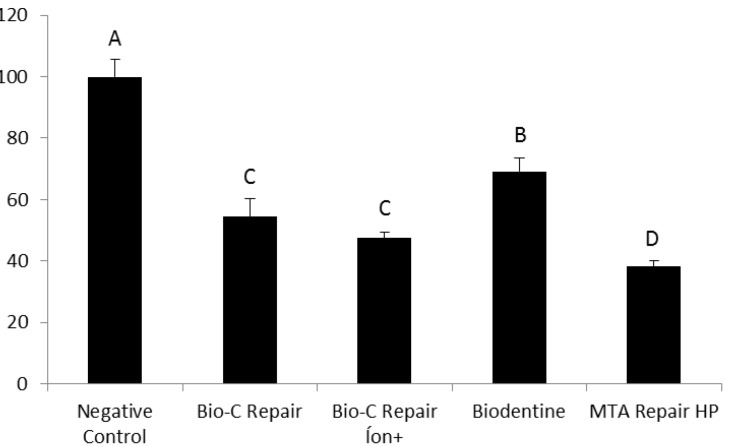
Cell viability of calcium silicate-based cements in the 7-day analysis.

All the tested calcium silicate-based cements resulted in a significant reduction in cell viability compared to the negative control (*P*≤0.001), as shown in Figures 1 and 2. The Biodentine, Bio-C Repair, and Bio-C Repair Íon+ exhibited lower cytotoxic effects than MTA Repair HP in the 24-hour analysis. All the tested calcium silicate-based cements resulted in a significant reduction in cell viability compared to the negative control, as shown in Figures 1 and 2. The Biodentine, Bio-C Repair, and Bio-C Repair Íon+ exhibited lower cytotoxicity than MTA Repair HP in the 24-hour analysis. In the 7-day analysis, Biodentine showed the highest cell viability, while MTA Repair HP was the most cytotoxic to NIH 3T3 cells. For this analysis period, the other calcium silicate-based cements exhibited moderate cytotoxicity, with no significant difference.

According to ISO 10993-5: 2009 (E) (Table 3), in the 24-hour analysis, Bio-C Repair, Biodentine, and Bio-C Repair Íon+ demonstrated mild cytotoxicity, while MTA Repair HP exhibited moderate cytotoxicity. In the 7-days analysis, Biodentine and Bio-C Repair displayed mild cytotoxicity, while Bio-C Repair Íon+ and MTA Repair HP exhibited moderate cytotoxicity.

**Table 3:** Classification of cytotoxicity of calcium silicate-based cements according to ISO 10993-5:2009 (E)

**Groups**	**24 hours**		**7 days**	
	**% Cell viability reduction**	**Toxicity classification**	**% Cell viability reduction**	**Toxicity classification**
Negative control	0	None	0	None
Bio-C Repair	31.65	Mild	45.55	Mild
Bio-C Repair Íon+	41.27	Mild	52.47	Moderate
Biodentine	38.53	Mild	30.78	Mild
MTA Repair HP	56.72	Moderate	61.80	Moderate

## Discussion

One of the significant challenges of a restorative technique and pulp therapy procedure is the process of tissue repair when it accidentally occurs, as in cases of traumatic or accidental pulp exposure and root perforation.^21^ The decrease in cell viability displayed by the materials was already expected since it is known that clinically direct pulp protection materials cause superficial necrosis in the tissue. This aggression results in cell differentiation and induction of the formation of a mineralized barrier.^22^ In the present *in vitro* analysis, all the materials exhibited a cytotoxic behavior due to a reduction in cell viability greater than 30% (ISO-10993-5: 2009 (E)),^23^ with the MTA group showing the greatest aggressiveness at both analysis intervals.

In the histological analysis in a clinical trial of exposed pulps using Biodentine and MTA, no inflammatory response and mineralized barrier formation were reported.^14^ Also, in another randomized clinical trial, Brizuela et al^24^ reported that calcium hydroxide, MTA, and Biodentine showed a high success rate in direct pulp capping, with no significant difference between the groups. It should be noted that Biodentine did not exhibit any failure in the 25 teeth treated and followed for one year. In the present study, this material exhibited the least cellular aggression over time, and this might be one of the reasons for its success in studies by Nowicka and Brizuela.^14,24^

Benetti et al^17^ reported that like MTA Repair HP, Bio-C Repair is biocompatible and has a biomineralization capacity when in contact with living tissues. In the same study’sin vitroanalysis, both materials exhibited similar cell viability on L929 fibroblasts. However, in the present study on NIH 3T3 fibroblasts, the bioceramic material exhibited cell viability superior to the mineral aggregate trioxide. This difference between the results can be explained by studies using different types of cells, each of which reacts differently to the materials.^25^

Cellular characteristics and functions are used to analyze and investigate the cytotoxicity of materials. In the present study, we verified rat fibroblasts’ behavior due to changes in the Stanford parameters. These cells have a type of cells present in the pulp tissue and gingival tissues due to their reproducible growth rates, advantages in handling, and availability compared to primary cells, in addition to being immortal cells.^26^ The cellular infeasibility presented by the MTT test does not exactly mean that there is a high rate of apoptosis and tissue necrosis, but that, in addition to these events already mentioned, there might also be a higher number of cells that have reduced metabolic activity.^27^

The use of metal ions, proposed by the Bio-C Repair Íon+, aims to increase the material’s bioactivity, mainly influencing the proliferation and cell differentiation to form mineralized tissue.^28,29^ In the present study, the version of the material that contains these ions in its composition demonstrated cell viability lower than the original version. The result is due to the presence of metal ions, as according to Mohammadi et al,^18^ high doses of these ions can cause toxic effects to cells. Besides, as the methodology used assesses the action of material extracts directly on cells in culture, it is impossible to obtain information regarding the bioactivity promoted by the material.

It is imperative for the material used in contact with the pulp tissue to have good biocompatibility and bioactive effects; otherwise, it will irritate the pulp, with a greater possibility that it will be irreversible. In vivoinvestigations are necessary to prove the good biological and mechanical properties of the new bioceramics materials. The present in vitro study demonstrated the cytocompatibility of materials used for direct pulp protection by analyzing cell viability.

## Conclusion

Considering some limitations of the present study, all the materials resulted in a reduction in cell viability. Bio-C Repair Íon+ and Bio-C Repair demonstrated moderate cytotoxicity, while Biodentine and MTA Repair HP exhibited the highest and the lowest toxicity to NIH 3T3 cells, respectively.

## Authors’ Contributions

Conceptualization: CAKJ, RZ, and EGR. Methodology: TD, VO, and DRM. Data Curation: CAKJ, RZ, and EGR. Writing the original draft: CAKJ, TD, and VO. Writing, review, and editing: RZ, EGR, and AÖ. All the authors have read and agreed to the final version of the manuscript.

## Acknowledgments

Not applicable.

## Funding

Not applicable.

## Competing Interests

The authors declare no competing interests with regards to the authorship and/or publication of this article.

## Ethics Approval

Not applicable.
